# Increased Secretory Leukocyte Protease Inhibitor (SLPI) Production by Highly Metastatic Mouse Breast Cancer Cells

**DOI:** 10.1371/journal.pone.0104223

**Published:** 2014-08-11

**Authors:** Kevin T. Sayers, Alan D. Brooks, Thomas J. Sayers, Oleg Chertov

**Affiliations:** Cancer Research Technology Program, Leidos Biomedical Research, Inc., Frederick National Laboratory for Cancer Research, Frederick, Maryland, United States of America; Cancer and Inflammation Program, Leidos Biomedical Research Inc., Frederick National Laboratory for Cancer Research, Frederick, Maryland, United States of America; Carl-Gustav Carus Technical University-Dresden, Germany

## Abstract

The precise molecular mechanisms enabling cancer cells to metastasize from the primary tumor to different tissue locations are still largely unknown. Secretion of some proteins by metastatic cells could facilitate metastasis formation. The comparison of secreted proteins from cancer cells with different metastatic capabilities *in vivo* might provide insight into proteins involved in the metastatic process. Comparison of the secreted proteins from the mouse breast cancer cell line 4T1 and its highly metastatic 4T1.2 clone revealed a prominent differentially secreted protein which was identified as SLPI (secretory leukocyte protease inhibitor). Western blotting indicated higher levels of the protein in both conditioned media and whole cell lysates of 4T1.2 cells. Additionally higher levels of SLPI were also observed in 4T1.2 breast tumors in vivo following immunohistochemical staining. A comparison of SLPI mRNA levels by gene profiling using microarrays and RT-PCR did not detect major differences in SLPI gene expression between the 4T1 and 4T1.2 cells indicating that SLPI secretion is regulated at the protein level. Our results demonstrate that secretion of SLPI is drastically increased in highly metastatic cells, suggesting a possible role for SLPI in enhancing the metastatic behavior of breast cancer cell line 4T1.

## Introduction

Secreted proteins have been shown to play an important role in the tumor metastasis of numerous cancers including breast [1], ovarian [2], lung [3], and a number of others. The secretion of certain proteins has been shown to be related to the aggressiveness of cancer cell growth and the ability of the cancer cells to metastasize. Tumor secreted proteins are involved in a number of biological processes including changes to the extracellular matrix [4] [5], angiogenesis [6], migration of cancer cells [7], and more recently a potential involvement in epithelial to mesenchymal transition (EMT) of cancer cells [8]. The investigation of secreted proteins can therefore provide an important insight into factors which might contribute to primary tumor growth and subsequent metastasis to other tissue sites.

The mouse breast cancer cell line 4T1 has been used to model human breast cancer since it demonstrates a similar disease progression to that seen in humans [9]. A clone of the 4T1 cell line called 4T1.2 has been isolated that metastasizes readily to lungs and bone with micrometastases often seen in other tissues such as axillary lymph nodes, heart, adrenal glands, rib cage and occasionally liver. Following orthotopic injection into the mammary fat pad 4T1.2 cells spontaneously metastasizes to bone to a much greater extent than the parental 4T1 cells, and this closely parallels the disease progression often observed in breast cancer patients [9]. Thus the 4T1.2 model has been used extensively to study breast cancer metastasis [10]. Therefore differences in secreted proteins between these two cell lines might be indicative of proteins that could be involved in the metastatic processes.

Though the number of secreted proteins is considerably lower than intracellular proteins hundreds of proteins could still be secreted by cells in cell culture. Therefore effective analysis of cell conditioned media must rely on biochemical fractionation of these proteins. A number of fractionation techniques have previously been used to analyze secreted proteins from cell lines including two dimensional electrophoresis (2-D PAGE) [11], one dimensional SDS-PAGE [12], and different chromatographic techniques including online and offline HPLC coupled with mass spectrometry [13], [14]. Mass spectrometry has been used previously to identify proteins secreted preferentially by cancer cells [15]. Here we used a combination of ion-exchange chromatography and SDS-PAGE to detect proteins which were differentially secreted by these two breast cancer cell lines that exhibit different metastatic properties.

## Materials and Methods

### Generation of conditioned media and cell lysates from 4T1 and 4T1.2 cells

The 4T1 cells were purchased from the American Type Culture Collection (ATCC, Manassas, VA). The 4T1.2 cell line was kindly provided by Dr. Mark Smyth, Peter MacCallum Cancer Center, East Melbourne, Australia). Multiple samples from the cell lines were used to minimize the impact of cell culture conditions and number of cell passages on the results obtained. Initially the cells were grown in complete RPMI media (RPMI, 5% FBS, Pen-strep (100 units/ml), NEAA (1x), HEPES (10 mM), Glutamax (2 mM), Sodium Pyruvate (1 mM), 2ME (5×10^−5^M). Cells were passed weekly at 1∶100 and 1∶400 dilutions. Conditioned media (CM) generation was performed using flasks of cells that were between 80–95% confluent. The media was removed, and the cells were then gently washed 3 times with warm PBS and the media was replaced with complete media lacking FBS. The cells were incubated in the FBS free media overnight and then the CM were decanted and cells were pelleted by centrifugation at 16000 g for 15 minutes. The CM were collected and filtered through 0.45 µm filters and used immediately or stored at −20°C.

Additional CM were produced by plating 2×10^6^ cells per well in a 6 well plate in serum-containing complete media (1 mL) and incubated overnight. CM were collected and centrifuged as previously described. For the preparation of cell lysates, after three washes with PBS, the cells were lysed by the addition of 250 µL of superlysis buffer (50 mM Tris-HCl pH 8.0, 0.5% Triton X-100, 300 mM NaCl, 5 mM EDTA plus Pierce complete protease inhibitor) to each well. The plate was placed on a shaker for 3 min then the remaining attached cells were scraped using a cell scraper and transferred to 1.5 mL tubes. The tubes were incubated on ice for 40 min, and then centrifuged at 16000 g for 15 minutes. Supernatants were removed and protein concentration was determined using a BCA Protein Assay Kit (Thermo Scientific).

### Two dimensional biochemical separation of secreted proteins

Proteins from the CM prepared in the absence of serum were separated using a combination of anion exchange chromatography and SDS-PAGE resulting in a two dimensional separation of the proteins. A gravity fed DEAE-650M anion exchange column 1.6 cm×1.5 cm was equilibrated in PBS buffer diluted three fold with water (1/3 PBS). A 20 mL volume of each supernatant was diluted with 40 mL of water resulting in the same salt concentration as the equilibrating buffer and loaded onto the column. Then column was washed with 10 mL of 1/3PBS. Elution was done stepwise with 800 µL of 0.1 M, 0.2 M, and 0.5 M NaCl in water. Each fraction was precipitated using one volume of trichloroacetic acid (TCA) (1.42 g/mL) to four volumes of sample fraction, and allowed to precipitate overnight at −20°C. The samples were then centrifuged in table top Eppendorf centrifuge at 16000 g to pellet the proteins. The pellets were washed twice with cold acetone and the precipitated proteins were dissolved in 20 µL of 1x SDS-electrophoresis loading buffer and loaded equally on two gels (10% Tricine, Invitrogen). One gel was stained with Coommassie Brilliant Blue and the other was used for electroblotting of proteins to PVDF membrane, Coommasie staining and N-terminal sequence analysis.

### Identification of differentially expressed proteins

Differentially stained bands were excised from the gel and digested by trypsin according to the method described by Shevchenko et al. [16]. The peptides were purified using µ-C-18 ZipTip (Millipore), and analyzed using a Bruker Ultraflex-III MALDI-TOF MS/MS with α-cyano-4-hydroxycinnamic-acid (CHCA) as matrix. MALDI-TOF spectrum data was processed by Bruker flexAnalysis (version 3.3). The spectra information was then exported to BioTools (version 3.34) and submitted to the MASCOT MS/MS Ion Search. The search resulted in a confident identification of SLPI, and the MASCOT result was imported back into BioTools to annotate the spectrum. Both NCBInr and SwissProt databases were searched with the following parameters partials < = 1, mass tolerance MS 0.1 Da, global modifications carbamidomethyl, enzyme trypsin, and organism Mus Musculus.

In addition, proteins from the SDS-PAGE were also transferred onto a PVDF membrane, and then stained with Coomassie brilliant blue. Protein bands were excised and analyzed by N-terminal Edman sequencing using an Applied Biosystems 494 cLC Protein sequencer. The amino acid phenylthiohydantoin derivatives were analyzed on-line using an Applied Biosystems 785A/140C/610A chromatograph.

### Western Blotting of conditioned media and cell lysates

Serum-free CM from each of the cell lines was precipitated for analysis by Western blotting. A 1.5 mL aliquot of the CM was mixed with four volumes of chilled acetone, the samples were allowed to precipitate overnight at −20°C. The protein in each of the samples was then pelleted by centrifugation at 16000 g. Then precipitated protein was dissolved in 40 µL of 1x loading buffer containing 6 mM DTT and 25 µL was loaded onto the gel. For cells grown in the presence of FBS, 35 µL of CM was mixed with 9 µL of 5x loading buffer containing DTT. Cell lysates were analyzed using the same conditions as for CM, with 20 µg of total protein loaded per well. After boiling for 10 minutes the samples were loaded onto the gel (4–20% mini-PROTEAN TGX, Bio-Rad). Transfer was carried out using a Trans-Blot Turbo (Bio-Rad) blotter on turbo settings for 1 mini-PROTEAN TGX gel. The membrane was washed twice in methanol and allowed to dry before being rehydrated with water. The membrane was then rinsed briefly with wash buffer (20 mM Tris buffered saline +0.5% Tween 20, pH 7.4), and incubated for two hours in blocking buffer (Tris buffered saline +0.5% Tween 20+1% nonfat dried milk). The SLPI primary antibody (R&D Systems mSLPI Goat IgG AF1735) was made up 1∶1000 in blocking buffer and incubated overnight at 4°C. The membrane was washed, and the secondary antibody (Pierce Mouse Anti-Goat IgG 31400) was used at 1∶50,000 with incubation for 1 hour at room temperature on a rocker. The membrane was washed and subsequently developed using the SuperSignal West Dura Substrate (Thermo Scientific). Kodak BioMax MR film was used for detection of signal at various exposure times.

### Detection of SLPI by immunohistochemistry

For the generation of tumors in vivo, female Balb/c mice (7–12 weeks of age) bred at the NCI Frederick were injected with 10^5^ 4T1 or 4T1.2 cells into the mammary fat pad. Mice were monitored daily for any signs of distress. When tumors reached approximately 100 mm^3^ in volume mice were painlessly euthanized with CO_2_ to effect from a house line as outlined in the Frederick National Laboratory for Cancer Research Animal Care and Use Committee (ACUC) guidelines. Frederick National Laboratory for Cancer Research is accredited by AAALAC International and follows the Public Health Service Policy for the Care and Use of Laboratory Animals. Animal care was provided in accordance with the procedures outlined in the "Guide for Care and Use of Laboratory Animals" (National Research Council; 1996; National Academy Press; Washington, D.C.).

Cell pellets from 4T1 and 4T1.2 passed in vitro, as well as the in vivo breast tumors, were fixed in 10% neutral-buffered formalin, routinely processed, embedded in paraffin and sectioned at 5 µm. Antigen retrieval was performed in citrate buffer (BioGenex) using Biocare's Decloaking Chamber. Endogenous peroxidase activity was quenched with a 15-minute incubation in 0.6% H_2_O_2_ in methanol. Following a 5% normal-goat-serum block, secretory leukocyte peptidase inhibitor antibody (SLPI; 1∶7500, rabbit polyclonal, Origene) was applied overnight at 4°C. Biotinylated secondary goat anti-rabbit, then Vector's Elite Standard ABC kit were utilized with detection by diaminobenzidine (DAB). Normal rabbit isotype was used for the negative control.

### Gene expression profiling

Gene expression was determined using an Affymetrix Mouse Exon 1.0 ST gene array. Total RNA was extracted from both of the cell lines using the Invitrogen mirVana miRNA isolation kit. Analysis of the array data was performed by the CCRIFX bioinformatics group of the Frederick National Laboratory.

### RT-PCR

RNA from the two cell lines was isolated using the Invitrogen TRIzol Plus RNA purification system. The cDNA synthesis was performed on the isolated RNA using the SuperScript III First Strand Synthesis System (Invitrogen) following the kit instructions, and using random hexamer primers. The cDNA was then amplified using HotStartTaq Plus Master Mix Kit (Qiagen), with the forward (TGATCCTCGGGACTGGTCAT) and reverse (ATCGGTGAATGCTGAGCCAA) primers for SLPI. The SLPI cDNA was amplified using a Verti Thermal Cycler (Applied Biosystems) set for 35 cycles with a reannealing temperature of 55°C.

## Results

### 4T1.2 cells secrete increased amount of a protein identified as SLPI

**Figure 1:**
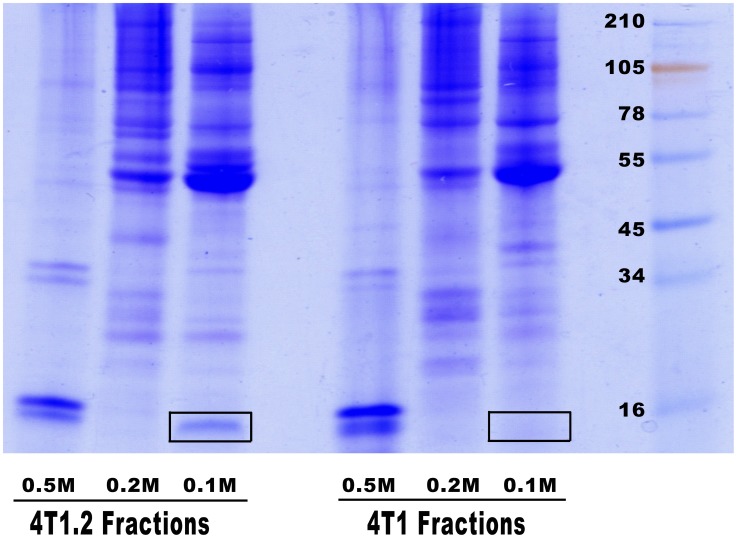
Proteins secreted by 4T1 and 4T1.2 cells. Cell conditioned media generated under serum-free conditions was fractionated on DEAE column and proteins were eluted step-wise by solutions containing 0.1 M, 0.2 M and 0.5 M of sodium chloride. After precipitation aliquots of fractions were separated on SDS gel and stained with Coomassie Brilliant blue for visual comparison of protein bands. Selected differences observed between the two cell lines are highlighted.

**Figure 2:**
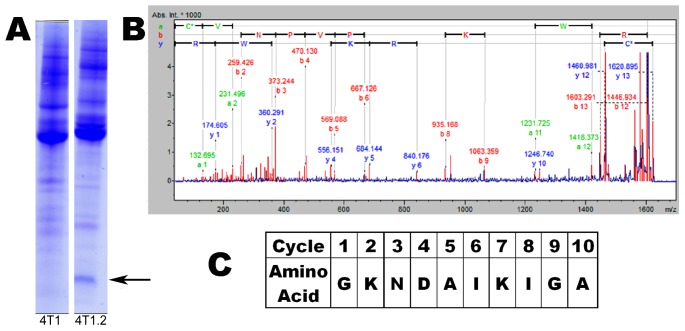
Identification of SLPI as a protein secreted at higher levels by 4T1.2 cells. (A). Coomassie stained SDS-PAGE gel of 0.1 M eluate from DEAE column with a difference in a protein of approximately 12 kDa indicated. (B). The band was excised and in-gel trypsin digestion was performed, a tryptic peptide with m/z of 1620.914 Da was analyzed using MALDI-TOF MS/MS and the resulting peptide mass spectrum was searched using MASCOT giving an identification of the peptide CVNPVPIRKPVWR from SLPI. (C). N-terminal Edman sequencing of the first 10 residues when searched with BLAST also indicated the band was SLPI.

A comparison of the secreted proteins from the mouse breast cancer cell lines 4T1 and 4T1.2 was carried out to identify proteins associated with the highly metastatic 4T1.2 phenotype. Proteins secreted by 4T1 and 4T1.2 were fractionated using anion exchange chromatography on DEAE-650 with step-wise elution by 0.1, 0.2 and 0.5 M NaCl. The eluted proteins were separated by SDS gel electrophoresis. The Coomassie stained gel was used to visually compare protein bands. Most protein bands observed from CM of 4T1 and 4T1.2 cells were identical and of similar intensity, but one standout protein band was predominantly present in 0.1 M eluate of 4T1.2 cell supernatant (**Fig.1** and **Fig.2A**). The band on a Coomassie stained gel was digested with trypsin and the obtained peptides analyzed by MALDI-TOF MS/MS. The spectrum of a peptide with m/z of 1620.914 Da was searched using MASCOT MS/MS Ion Search which resulted in confident identification of the peptide CVNPVPIRKPVWR of SLPI using both SwissProt (e = 1.7×10^−3^) and NCBInr (e = 2.7×10^−4^) databases (**Fig. 2B**). Analysis of m/z 1276.572 allowed identification of sequence TDWECPGKQR of SLPI (not shown).

This protein band when transferred to a PVDF membrane was analyzed by N-terminal protein sequencing. The sequence GKNDAIKIGA was established (**Fig.2C**). A Protein BLAST search of *Mus Musculus* proteins database resulted in complete match of this sequence to N-terminal sequence of mature Secretory Leukocyte Protease Inhibitor (SLPI_mouse). Determination of only one major sequence in this band by semi-quantitative Edman sequencing suggested that SLPI was a major component of this band and therefore SLPI is likely to be differentially produced by 4T1 and 4T1.2 cells.

### Western blot confirms increased SLPI secretion by 4T1.2 cells

**Figure 3:**
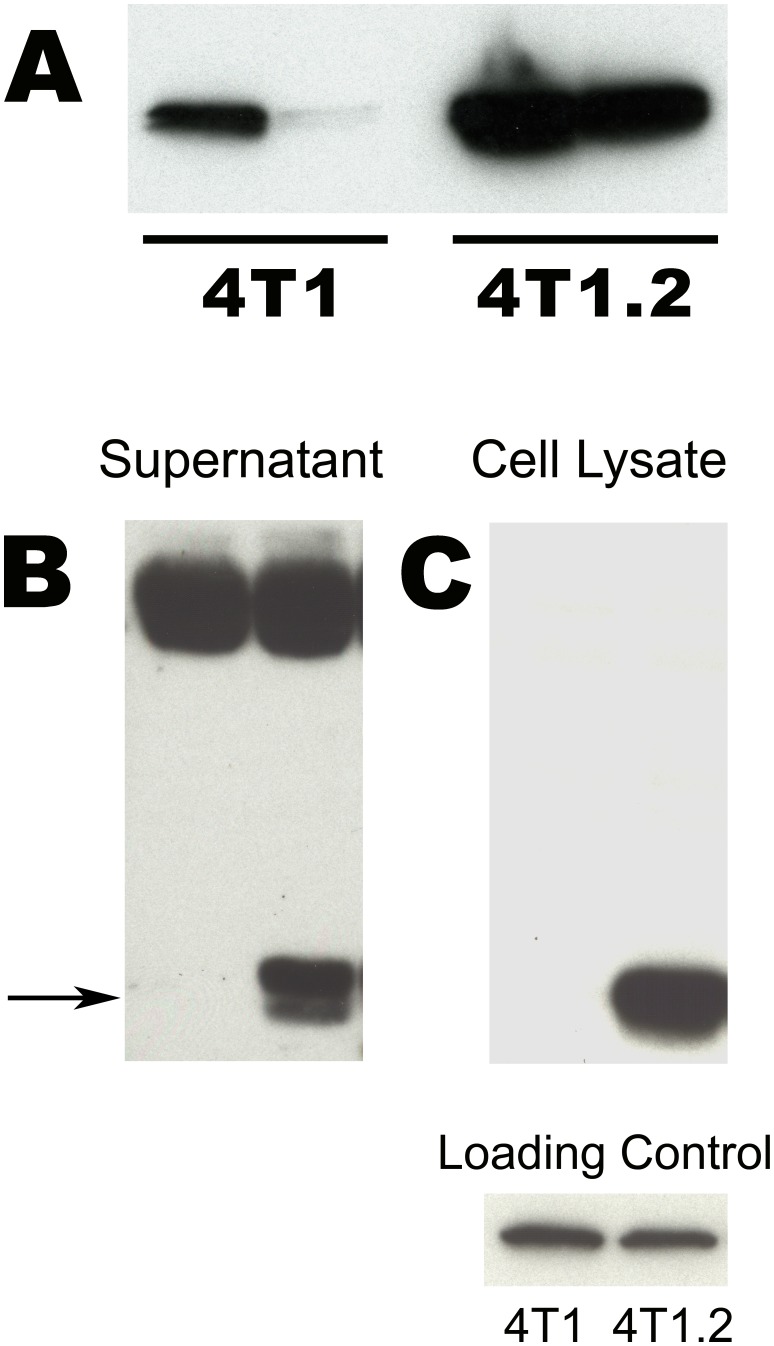
Increased production of SLPI by 4T1.2 cells. (A). Initial Western blot of CM generated in serum-free media to confirm differences in SLPI levels. Cell conditioned media preparations for both 4T1 and 4T1.2 from 2 separate experiments were loaded on the gel. SLPI levels were higher in 4T1.2 conditioned media. (B). Western blot for SLPI of CM from cells grown in media containing FBS. The 4T1.2 CM clearly had increased levels of SLPI as compared to the 4T1 conditioned media. Also observed was a high molecular weight non-specific band believed to be BSA which indicates comparable overall protein loading of CM on these gels. (C). Western blot of cell lysates showing intracellular SLPI was also highly elevated in 4T1.2 cells. Reprobing of the cell lysate blot with an antibody to actin confirms equivalent loading of proteins on the gel.

Confirmation of the differences in SLPI secretion between the 4T1 and 4T1.2 cell lines was performed using Western Blotting with an SLPI antibody. Comparison of band intensities between the 4T1 and 4T1.2 samples indicated that SLPI was present at much higher levels in the 4T1.2 CM (**Fig. 3B** **).** This increased intensity of the SLPI band in 4T1.2 CM samples was consistently observed using multiple CM preparations. The Western blots were repeated with CM of cells cultured with serum-containing media, and under these conditions even greater differences in SLPI levels between the 4T1 and 4T1.2 CM were observed (**Fig. 3B** **)**. In an attempt to determine if the higher level of SLPI present in the CM was due to differential secretion of SLPI or overall higher total protein levels, analysis of the cell lysates was also carried out. Western blots of cell lysates mirrored what was observed for the CM, with 4T1.2 lysates having significantly higher levels of SLPI (**Fig. 3C** **)**. The cell lysate blot was re-probed for actin to show that the amount of protein present on the blot was comparable (**Fig. 3C**). Since Western blotting of the cell lysates for intracellular SLPI between 4T1 and 4T1.2 showed similar differences as was observed for secreted proteins, the results indicated that much higher overall levels of SLPI were produced by the 4T1.2 cell line as compared with 4T1.

### Detection of SLPI by immunohistochemistry

**Figure 4:**
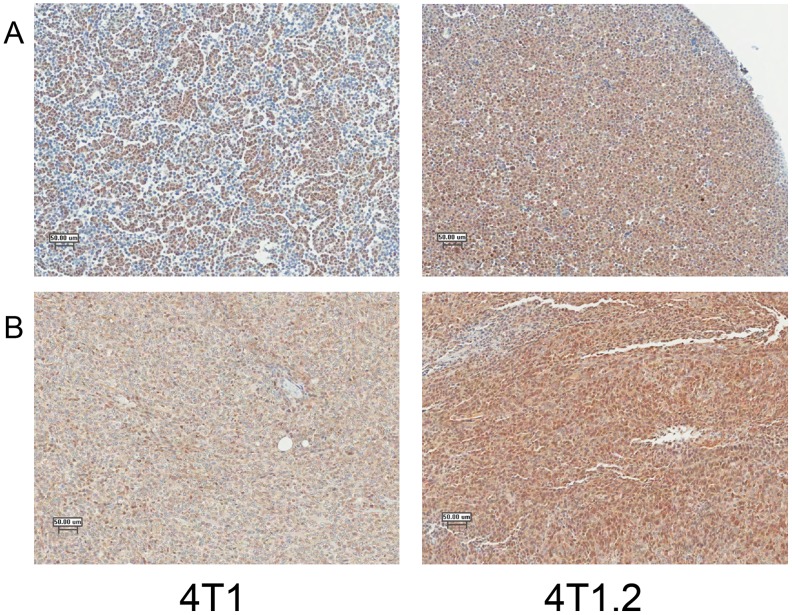
Staining of 4T1 and 4T1.2 cells for SLPI. Immunohistochemical staining also indicates increased production of SLPI by 4T1.2 cells. (A). Staining of cell pellets of 4T1 and 4T1.2 cells grown in vitro shows that almost all 4T1.2 cells produce SLPI whereas production by 4T1 cells is confined to a subpopulation of cells. (B). Staining of breast tumors derived from the injection of 4T1 and 4T1.2 cells also demonstrates a stronger and more uniform staining of SLPI in the 4T1.2 tumors.

Staining of 4T1 and 4T1.2 cell pellets (**Fig. 4A**) as well as tumors derived following injection of these cells into the mammary fat pad of mice (**Fig. 4B**) both demonstrated increased staining for SLPI in 4T1.2 samples. Interestingly for both the cell pellets and tumors, the major difference was in the percentage of cells staining positive for SLPI (much higher in 4T1.2 cells) rather than differences in staining intensity of SLPI positive cells. Since the 4T1.2 clone was originally derived from the 4T1 cell line, the SLPI staining of some cells in the 4T1 samples may be accounted for by the presence of individual clones similar or identical to 4T1.2.

### SLPI gene expression is the same in 4T1 and 4T1.2 cells

In an attempt to determine if the differences in SLPI protein expression were associated with differences in mRNA levels, RT-PCR and genechip microarray experiments were performed. Surprisingly the genechip microarray analysis showed no differences in gene expression between the 4T1 and 4T1.2 lines. Furthermore the mRNA levels in the 4T1 and 4T1.2 cells observed from the RT-PCR using SLPI primers were nearly identical (data not shown).

## Discussion

Identification of differentially secreted proteins from highly metastatic cancer cells might further aid in our understanding of the underlying molecular mechanisms of metastasis. Furthermore proteins which are determined to be important for metastasis and cancer cell survival could also have the potential of being important targets in cancer therapy [17]. When comparing 4T1 and 4T1.2 cell lines we found a clear increase in both the production and secretion of SLPI by the more metastatic 4T1.2 cell line. This increased SLPI level was not only observed in cells grown in tissue culture media, but also in breast carcinomas generated from these cells in vivo.

Although major differences were observed in intracellular and secreted levels of SLPI in the 4T1 and 4T1.2 cells, analysis of mRNA from these cell lines did not detect any significant differences. Thus a careful biochemical analysis of secreted proteins revealed differences in SLPI levels that would not have been predicted from gene microarray data. These differences in the levels of SLPI protein, despite similar levels of RNA, may be indicative of a post-transcriptional control of SLPI. Regulation of SLPI by miRNA has previously been proposed [18], and may thus explain the drastic difference between mRNA and protein levels. Interestingly a preliminary analysis of miRNA levels in 4T1 and 4T1.2 cells revealed substantial differences in the levels of a number of miRNA (data not shown). Therefore the potential control of SLPI by miRNA is worthy of further investigation.

A number of recent studies have described a role for SLPI in tumor development and progression. Elevated gene expression of *SLPI* has been associated with cancer cell growth and metastasis in ovarian cancer [19]. Overexpression of SLPI in lung cancer cells was shown to lead to a more malignant phenotype [20]. Furthermore, SLPI has been proposed to protect cells from anoikis, a form of cell death which follows loss of adhesion [19].

It has also been reported that SLPI protects the protein progranulin from enzymatic cleavage [19]. Like SLPI, progranulin has been associated with aggressive tumorigenesis [21], [22] by promoting invasiveness of breast cancer cells [22]. Gene microarray profiling for progranulin showed higher levels of progranulin mRNA in 4T1 cells, yet we determined that the level of secreted progranulin was significantly elevated in 4T1.2 CM by ELISA (data not shown), supporting a possible protective role of SLPI in maintaining progranulin activity.

In conclusion, we used two-dimensional biochemical separation to compare the secreted proteins from the mouse breast cancer cell line 4T1 and 4T1.2 clone which has been previously shown to be more metastatic. We identified significantly elevated levels of the secreted protein SLPI in more metastatic cell line 4T1.2 despite insignificant difference in mRNA levels. The difference between mRNA and protein levels of SLPI that we observed in these breast cancer cells illustrate that care must be taken when interpreting gene microarray data, and it is important to perform additional biochemical analysis when analyzing proteins that could be involved in cancer cell survival. High levels of SLPI have been previously associated with enhanced tumor progression. Further work is necessary to determine the biological role of SLPI in breast cancer progression to metastasis.
